# Author Correction: A novel methodology for epidemic risk assessment of COVID-19 outbreak

**DOI:** 10.1038/s41598-021-94234-0

**Published:** 2021-07-28

**Authors:** A. Pluchino, A. E. Biondo, N. Giuffrida, G. Inturri, V. Latora, R. Le Moli, A. Rapisarda, G. Russo, C. Zappalà

**Affiliations:** 1 Dipartimento di Fisica e Astronomia “Ettore Majorana”, INFN Sezione di Catania, Università di Catania, Catania, Italy; 2 Dipartimento di Economia e Impresa, Università di Catania, Catania, Italy; 3 Dipartimento di Ingegneria Civile e Architettura, Università di Catania, Catania, Italy; 4 Dipartimento di Ingegneria Elettrica Elettronica e Informatica, Università di Catania, Catania, Italy; 5 Complexity Science Hub Vienna, Vienna, Austria; 6 School of Mathematical Sciences, Queen Mary University of London, London, E1 4NS UK; 7 The Alan Turing Institute, The British Library, London, NW1 2DB UK; 8 Dipartimento di Medicina Clinica e Sperimentale ‑ UO di Endocrinologia ‑ Ospedale Garibaldi Nesima, Università di Catania, Catania, Italy; 9 Dipartimento di Matematica e Informatica, Università di Catania, Catania, Italy

Correction to: *Scientific Reports* 10.1038/s41598-021-82310-4, published online 05 March 2021

The original version of this Article contained an error in Figure 1a where the values shown in the key for ‘Case Fatality rate_2apr’ were incorrect.

The original Figure 1 and accompanying legend appear below.

**Figure 1:**
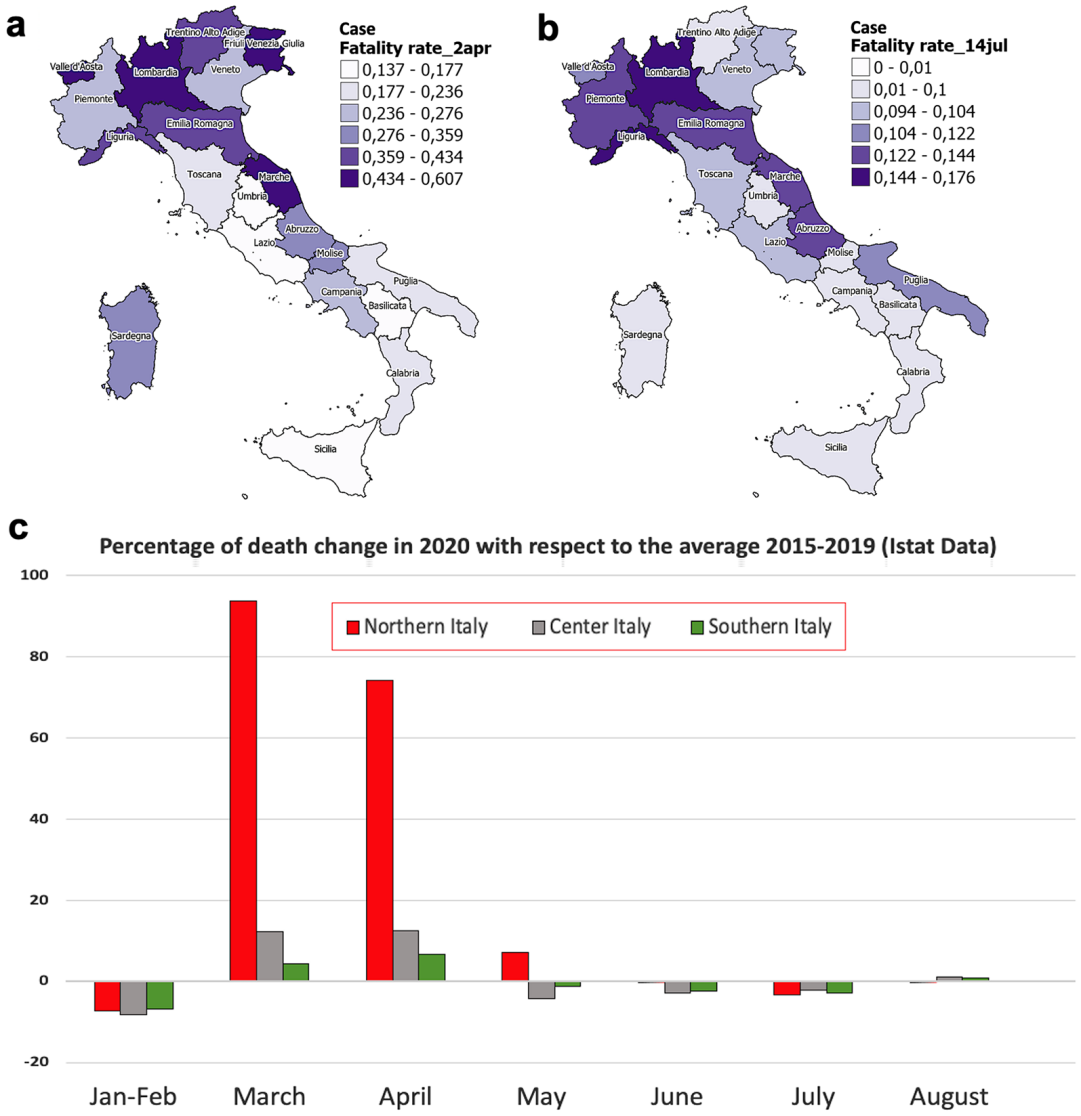
(**a**) Color geographic maps of the apparent case fatality rate in the various regions on April 2, 2020 and on July 14, 2020 (**b**), data released by the Italian Ministry of Health^4^. Maps were realized with QGIS 3.10 (https://qgis.org/en/site/). (**c**) Percentage of deaths change in 2020 with respect to the average taken in the period 2015–2019 (ISTAT^22^).

The original Article has been corrected.

